# Proteomic analysis of chicken bone marrow-derived dendritic cells in response to an inactivated IBV + NDV poultry vaccine

**DOI:** 10.1038/s41598-021-89810-3

**Published:** 2021-06-16

**Authors:** Robin H. G. A. van den Biggelaar, Larissa van der Maas, Hugo D. Meiring, Jeroen L. A. Pennings, Willem van Eden, Victor P. M. G. Rutten, Christine A. Jansen

**Affiliations:** 1 Division of Infectious Diseases and Immunology, Department of Biomolecular Health Sciences, Faculty of Veterinary Medicine, Utrecht University, Utrecht, The Netherlands; 2 Intravacc (Institute for Translational Vaccinology), Bilthoven, The Netherlands; 3 Centre for Health Protection, National Institute for Public Health and the Environment (RIVM), Utrecht, The Netherlands; 4 Department of Veterinary Tropical Diseases, Faculty of Veterinary Science, University of Pretoria, Pretoria, South Africa; 5 Cell Biology and Immunology Group, Department of Animal Sciences, Wageningen University and Research, Wageningen, The Netherlands

**Keywords:** Gene regulation in immune cells, Innate immune cells, Vaccines

## Abstract

Inactivated poultry vaccines are subject to routine potency testing for batch release, requiring large numbers of animals. The replacement of in vivo tests for cell-based alternatives can be facilitated by the identification of biomarkers for vaccine-induced immune responses. In this study, chicken bone marrow-derived dendritic cells were stimulated with an inactivated vaccine for infectious bronchitis virus and Newcastle disease virus, as well as inactivated infectious bronchitis virus only, and lipopolysaccharides as positive control, or left unstimulated for comparison with the stimulated samples. Next, the cells were lysed and subjected to proteomic analysis. Stimulation with the vaccine resulted in 66 differentially expressed proteins associated with mRNA translation, immune responses, lipid metabolism and the proteasome. For the eight most significantly upregulated proteins, mRNA expression levels were assessed. Markers that showed increased expression at both mRNA and protein levels included PLIN2 and PSMB1. Stimulation with infectious bronchitis virus only resulted in 25 differentially expressed proteins, which were mostly proteins containing Src homology 2 domains. Stimulation with lipopolysaccharides resulted in 118 differentially expressed proteins associated with dendritic cell maturation and antimicrobial activity. This study provides leads to a better understanding of the activation of dendritic cells by an inactivated poultry vaccine, and identified PLIN2 and PSMB1 as potential biomarkers for cell-based potency testing.

## Introduction

The safety and potency of poultry vaccines has traditionally been assessed through in vivo vaccination and challenge tests^1^. However, there is a global intent to replace in vivo vaccine tests for in vitro alternatives^2^. This has already led to the development of an enzyme-linked immunosorbent assay (ELISA) as an in vitro antigen quantification method to assess inactivated vaccines for Newcastle disease virus (NDV) for potency^3^. However, other inactivated poultry vaccines still require in vivo tests to prove their potency and are thus in need for in vitro alternatives. The development of in vitro tests for inactivated poultry vaccines can be facilitated by the identification of biomarkers that may be inherent to the vaccine, demonstrating a consistent product profile of different vaccine batches^4–6^, or be part of the vaccine-induced immune response that correlates with protection^7–10^. In particular, dendritic cells (DCs) are ideal study targets, since they act as professional antigen-presenting cells, responsible for activating naïve T cells upon vaccination^11^. DCs express many pattern recognition receptors (PRRs) that are able to detect pathogen-associated molecular patterns (PAMPs) associated with (inactivated) pathogens present in vaccines. Whether a vaccine evokes an immune response is ultimately determined by its capacity to activate DCs through PRRs, resulting in antigen-presentation and activation of naïve T cells. One approach to discover biomarkers of immune responsiveness to inactivated poultry vaccines is the investigation of in vitro vaccine-stimulated dendritic cells by proteomic analysis using liquid chromatography-tandem mass spectrometry (LC–MS/MS).

Both infectious bronchitis virus (IBV) and NDV cause respiratory tract infections that can disseminate to other tissues, leading to reduced egg production and mortality in chickens^12,13^. In this study, primary chicken bone marrow-derived DCs (chBMDCs), which have been characterized in previous studies^14,15^, were stimulated with a commercially available inactivated poultry vaccine against IBV and NDV in a mineral oil adjuvant in a water-in-oil formulation, and analyzed by LC–MS/MS to evaluate changes in the proteome In addition, chBDMCs were stimulated with inactivated IBV antigens only to discriminate between the effects of a single inactivated antigen and a vaccine. Stimulation with *E. coli* lipopolysaccharide (LPS) was included in this study as a strong stimulator of innate immune cells to validate the methodology used. Previous studies have shown that LPS stimulates maturation of chicken DCs, including increased surface expression of MHC class II molecules and costimulatory molecules^14–19^, decreased endocytosis and phagocytosis^14,15^, increased expression of proinflammatory cytokines and cytokines that promote the differentiation of naïve helper T cells^14,17,19^, and an increased capacity to stimulate naïve T cells^14,15,18^. The primary aim of this study was to search for biomarkers of vaccine-stimulated DCs to use as targets for in vitro cell-based quality assessment of an inactivated IBV + NDV poultry vaccine. Secondly, this study may generate new hypotheses about the mechanisms by which an inactivated viral poultry vaccine activates chicken DCs, as well as new insights in the cellular processes involved in chBMDCs maturation after stimulation with LPS.

## Methods

### Generation and stimulation of chBMDCs

In this study, bone marrow was isolated from eighteen-day-old NOVOgen Brown embryos (Verbeek Broederij, Zeewolde, The Netherlands) as described previously^15^. All methods were conducted in accordance with protocols approved by the Department of Biomolecular Health Sciences, Division of Infectious Diseases & Immunology of the Utrecht University. Although according to European legislation for the protection of animals used for scientific purposes (Directive 2010/63/EU) experiments with embryonated chicken eggs are not considered animal experiments, the study was carried out in compliance with the ARRIVE guidelines. Briefly, the bone marrow cells were obtained from the tibiae and femurs of 200 chicken embryos, which were pooled and resuspended with 2.5–5 × 10^7^ cells/ml in Roswell Park Memorial Institute (RPMI)-1640 cell culture medium supplemented with GlutaMAX-I, phenol red, HEPES (RPMI-1640; Gibco, Life Technologies Limited, Paisley, UK), 50% chicken serum (Gibco) and 10% dimethyl sulfoxide (Honeywell, Bucharest, Romania). The cells were viably frozen with 2.5–5 × 10^7^ cells per cryotube and stored at − 140 °C until further use.

Frozen bone marrow cells were thawed and cultured in Costar 24-well plates (Corning, Corning B.V. Life Sciences, Amsterdam, The Netherlands) in 1 ml/well RPMI-1640 cell culture medium supplemented with 5% chicken serum and 50 U/ml of penicillin–streptomycin (all from Gibco) in the presence of 2 µl/ml recombinant GM-CSF at 41 °C, 5% CO_2_^15^. Recombinant GM-CSF was produced using COS-7 cells transfected with pCI-neo (Promega Corporation, Madison, WI, USA) expressing the relevant cytokine gene, a kind gift from P. Kaiser and L. Rothwell (Roslin Institute, Edinburgh, UK). GM-CSF was used at the concentration (2 μl/ml) that, after titration, resulted in the highest percentage of MHC-II^+^ CD40^+^ CD80^+^ cells, as determined using flow cytometry analysis^15^. In the morning of day 3, culture medium with non-adherent cells was removed, and 1 ml of fresh RPMI-1640 medium with GM-CSF was added. Approximately 32 h later (± 2 h), on day 4, an additional 1 ml RPMI-1640 medium with GM-CSF was added to the cultures. At day 7, chBMDCs were stimulated with 30 µl/ml IBV + NDV vaccine, 10 µl/ml IBV antigens (both were kind gifts from Boehringer Ingelheim, Ingelheim am Rhein, Germany), or 100 ng/ml of LPS from *Escherichia (E.)coli* O127:B8 (Sigma-Aldrich, Saint Louis, MO, USA) for 24 h, or left untreated. To create sample volumes with sufficient protein or RNA content, six wells of a 24-well plate were used for each condition (Supplementary Figure 1). After 24 h, the cell culture supernatant with floating cells was first collected. Subsequently, the cultures were washed with Dulbecco's phosphate-buffered saline without calcium and magnesium (PBS; Lonza, Basel, Switzerland) and the non-adherent cells were collected. The remaining adherent cells were incubated in PBS supplemented with 5 mM UltraPure EDTA (Invitrogen, Life Technologies Europe BV, Bleiswijk, The Netherlands) for 10 min at room temperature and harvested. All cell-containing fluids per culture condition (cell culture medium, PBS and PBS 5 mM EDTA) obtained during the harvest procedure were pooled for subsequent experiments. The samples were spun at 400 *g *for 5 min and the cells were resuspended in 100 mM phosphate buffer (Sigma-Aldrich) and stored at −80 °C for subsequent LC–MS/MS analysis or lysed in RLT buffer (QIAGEN GmbH, Hilden, Germany) and stored at −20 °C for RNA isolation. The preparation of chBMDC samples, for both the LC–MS/MS and gene expression analysis, was performed three times independently.

### Protein isolation, digestion and labelling

Frozen samples were thawed, and the cells were lysed in 100 mM phosphate buffer with 0.1% (w/v) RapiGest SF Surfactant (Waters Corporation, Milford, MA, USA) by vortexing and incubation at 80 °C for 30 min. Next, the protein content was determined by the Pierce BCA assay (ThermoFisher Scientific, Waltham, MA, USA) according to the manufacturer’s instructions. The proteins were digested with 0.05 mg/ml Lys-C (Roche, Merck, Kenilworth, NJ, USA) at an enzyme:substrate ratio of 1:400 for 4 h at 37 °C followed by digestion with 0.2 mg/ml trypsin (Promega) overnight at 37 °C at an enzyme:substrate ratio of 1:100. Next, aliquots from the digests were labelled with native formaldehyde (CH_2_O with *M* = 30.03 g/mol, hereinafter referred to as “light”) (Sigma-Aldrich) together with sodium cyanoborohydride (NaCNBH_3_) (Sigma-Aldrich), both at final concentrations of 30 mM, and incubation for 2 h at 37 °C. In addition, an internal standard was created by pooling digest aliquots of all stimulated samples (LPS, IBV antigen or IBV + NDV vaccine) and corresponding unstimulated samples, each of equal peptide mass as determined before digestion. These so-called “Common References” were labelled with deuterated formaldehyde (CD_2_O with *M* = 32.04 g/mol, hereinafter referred to as “heavy”) (Sigma-Aldrich), together with NaCNBH_3_, both at final concentrations of 30 mM, and incubation for 2 h at 37 °C.

### LC–MS/MS analysis and data processing

Each “light”-labelled sample was mixed with an aliquot of the “heavy”-labelled Common Reference in a 1:1 ratio (based on the mass of the peptides). The resulting mixtures were desalted on a GX-271 Automated Solid Phase Extraction system (Gilson International B.V., The Hague, The Netherlands). Briefly, each mixture was loaded onto a 1 ml capacity C18 cartridge (Waters) and the absorbed peptides were washed with 600 µl of water containing 0.1 vol% of formic acid and subsequently eluted with 600 µl of 60 vol% of acetonitrile in water, also containing 0.1 vol% of formic acid. Each eluate was dried by centrifugation under reduced pressure (Eppendorf Vacuum Concentrator Plus, Eppendorf, Germany) and reconstituted in 100 µl of water containing 5 vol% of dimethyl sulfoxide and 0.1 vol% of formic acid for *nano*scale LC–MS/MS analysis.

The *nano*scale LC–MS system comprised an Agilent 1290 Infinity UPLC system (Agilent, Waldbronn, Germany) coupled to a Tribrid Orbitrap Fusion Lumos mass spectrometer (ThermoFisher Scientific). The system was setup essentially as described^20^ containing an in-housemade trapping column (20 mm length × 100 µm I.D., Reprosil-Pur C18-AQ 5 µm particle size, 120 Å pore size, Dr. Maisch, Ammerbuch, Germany) and an analytical column (32.4 cm length × 50 µm I.D., Reprosil-Pur C18-AQ 3 µm particle size, 120 Å pore size, dr. Maisch GmbH) made in-house at Intravacc, The Netherlands. Solvent A was 0.1 vol% of formic acid in water and solvent B was 0.1 vol% of formic acid in acetonitrile. The peptides were trapped on the trapping column for 10 min at a flow rate of 5 µl/min of 100% solvent A. The subsequent separation of the peptides was performed at a flow rate of 125 nl/min in a 95-min non-linear gradient (8% solvent B to 28% solvent B in 0.25%/min, followed by an increase to 43% solvent B in 1.5%/min and a step to 85% solvent B hold for 5 min). The column effluent was electrosprayed into the MS with an in-housemade gold/carbon-coated fused silica spray tip (360 µm O.D. × 25 µm I.D., manually tapered to a 3.5-µm tip I.D.), butt-connected to the analytical column. Full scan mass spectra were acquired with an Orbitrap readout at a 120,000 FWHM resolution with an *m/z* scan range of 300–1,500 Da, a maximum ion injection time of 50 ms and the Automatic Gain Control (AGC) set to 200,000. Internal mass calibration with fluoranthene was enabled. CID fragmentation scans with an Ion Trap readout were acquired for *z* = 1 and *z* = 2–5 positively charged ions with intensity thresholds of 1 × 10^6^ or 5,000 counts, respectively. Dynamic exclusion was set to 2 for a 45-s time window. Default instrument settings were used for MS/MS acquisition.

Acquired data were processed with PEAKS X (Bioinformatics Solutions Inc., Waterloo, Canada) against the*Gallus gallus domesticus* database (Taxonomy ID = 9031, www.uniprot.org) containing 34,930 entries for protein identification. Enzyme specificity was set to trypsin (semispecific) with a maximum of 3 miscleavages. Mass error tolerances for parent ions and fragment ions were set to 20 ppm and 0.6 Da, respectively. Fixed modifications were carbamidomethylation on Cys (+ 57.0215 Da) and dimethylation on Lys (+ 28.0313 Da and + 32.0564 Da for "light" and "heavy", respectively). The variable modifications were deamidation on Asn and Gln (+ 0.9840 Da) and oxidation on Met (+ 15.9949 Da). Peak area intensities of the precursor ions of the identified peptides were used for relative protein quantification.

### Analysis of relative protein abundance

Data processing and statistical analysis was done using R statistical software, version 3.6.0^21^. The relative quantification of the samples was based on the comparison of all samples against the Common References (Supplementary Table S1) acting as internal standards between samples, in accordance with a previous publication^22^. The resulting sample / Common Reference ratios were Log_2_-transformed. Next, the values were normalized for variations between measurements by performing a median correction, in which all relative protein expression values were divided by the median protein expression value of each run (Supplementary Table S2), in accordance with previous publications^23,24^. Proteins were included as quantitative differentially expressed proteins (DEPs) if they were detected in at least two out of three replicates for both stimulated and unstimulated samples, if they showed an average upregulation or downregulation of at least 1.5-fold upon stimulation, and if this change was statistically significant (p < 0.05) according to a Student’s T-test. Moreover, de novo-expressed proteins (*i.e.* uniquely expressed proteins detected in all samples of the stimulated group and in no samples of the unstimulated group, or the reverse situation) were also included as qualitative DEPs in the results. Although the described statistics could not be performed for these de novo-expressed proteins with expression levels below the detection limit in one of the treatment groups, the qualitative differences were still considered to be of practical significance. Finally, some proteins appeared more than once in the results, while showing the same expression values. These redundant appearances were removed resulting in a list of unique proteins.

Benjamini–Hochberg corrections of *p*-values were performed to calculate the false discovery rates (FDR) of all proteins and an FDR < 0.1 was considered significant (Supplementary Table S3) ^28^. However, the sample size was too small to identify many DEPs based on FDR and the inclusion criteria based on *p*-values and 1.5-fold change as described above were used for further analysis.

Volcano plots were made using Perseus software^25^ to visualize the selection of DEPs. The selected DEPs were further analysed for protein–protein association networks using the STRING database (www.string-db.org)^26^. The database was searched for physical or functional protein–protein interactions with a minimum required interaction score of 0.700 (high confidence) among the DEPs. The resulting STRING network clusters were functionally classified using annotations with the lowest Benjamini–Hochberg false-discovery rates from integrated Reactome (www.reactome.org), KEGG (www.kegg.jp), GO (www.ebi.ac.uk/QuickGo/), PFAM (www.pfam.xfam.org) and SMART (www.smart.embl-heidelberg.de) databases. Additionally, the STRING network was exported as tabular text output, and combined with protein expression data in Microsoft Excel. The resulting files were loaded in Cytoscape^27^ version 3.7.2 for visualization.

The subcellular localization of DEPs was determined based on GO terms for cellular component using QuickGO (https://www.ebi.ac.uk/QuickGO/). The search was limited to a selected number of gene ontology terms for cellular components listed in Supplementary Table S4. The UniProtKB database was used as a reference for chicken proteins by choosing taxon identifier 9031 (*Gallus gallus*). The DEPs of stimulated chBMDCs were tested for enrichment for cellular components as compared to the reference using a one-tailed binominal test. Next, the Benjamini–Hochberg method was used to control false discovery rates (FDR) as a result of multiple comparisons^28^. An FDR < 0.05 was considered significant.

To create KEGG pathway maps, the KEGG Mapper tool (http://www.genome.jp/kegg/mapper.html)^29^was used to modify the maps gga03050 (Proteasome—Gallus gallus [chicken]), gga01100 (Metabolic pathways—Gallus gallus [chicken]) and gga04142 (Lysosome—Gallus gallus [chicken]). Permission was granted by Kanehisa Laboratories to publish the KEGG pathway map images both in print and digital under the CC BY 4.0 open access license.

### Gene expression analysis

RNA isolation was performed with the RNeasy Mini Kit (QIAGEN) according to the manufacturer's instructions, including a DNase treatment step using the RNase-Free DNase Set (QIAGEN). Next, cDNA was prepared using the reverse transcriptase from the iScript cDNA Synthesis Kit (Bio-Rad Laboratories B.V., Veenendaal, The Netherlands) according to the manufacturer's instructions. RT-qPCRs were performed for several genes of the most significantly upregulated DEPs with primers listed in Supplementary Table S5 and SYBR Green Master Mix (both from Life Technologies). RT-qPCRs were performed with a CFX Connect and analyzed with the CFX Maestro software (both from Bio-Rad). All RT-qPCRs were evaluated for proper amplification efficiency (90–110%) using serial dilutions of reference cDNA obtained from the chicken macrophage-like cell line HD11 after 3 h stimulation with LPS. RT-qPCRs were performed in triplicate for every sample. Relative gene expression levels after stimulation of chBMDCs were normalized using the housekeeping gene *GAPDH* as the reference gene expressed as log2-transformed fold changes compared to unstimulated chBMDCs. Genes were considered significantly differentially expressed if at least two out of three stimulated replicates were upregulated or downregulated compared to unstimulated controls, the change was at least 1.5-fold after stimulation, and the difference was statistically significant (*p* < 0.05) according to a Student’s T-test.

## Results

### Differentially expressed proteins identified in chBMDCs stimulated with IBV + NDV vaccine did not show overlap with differentially expressed proteins after stimulation with IBV antigen only

**Figure 1:**
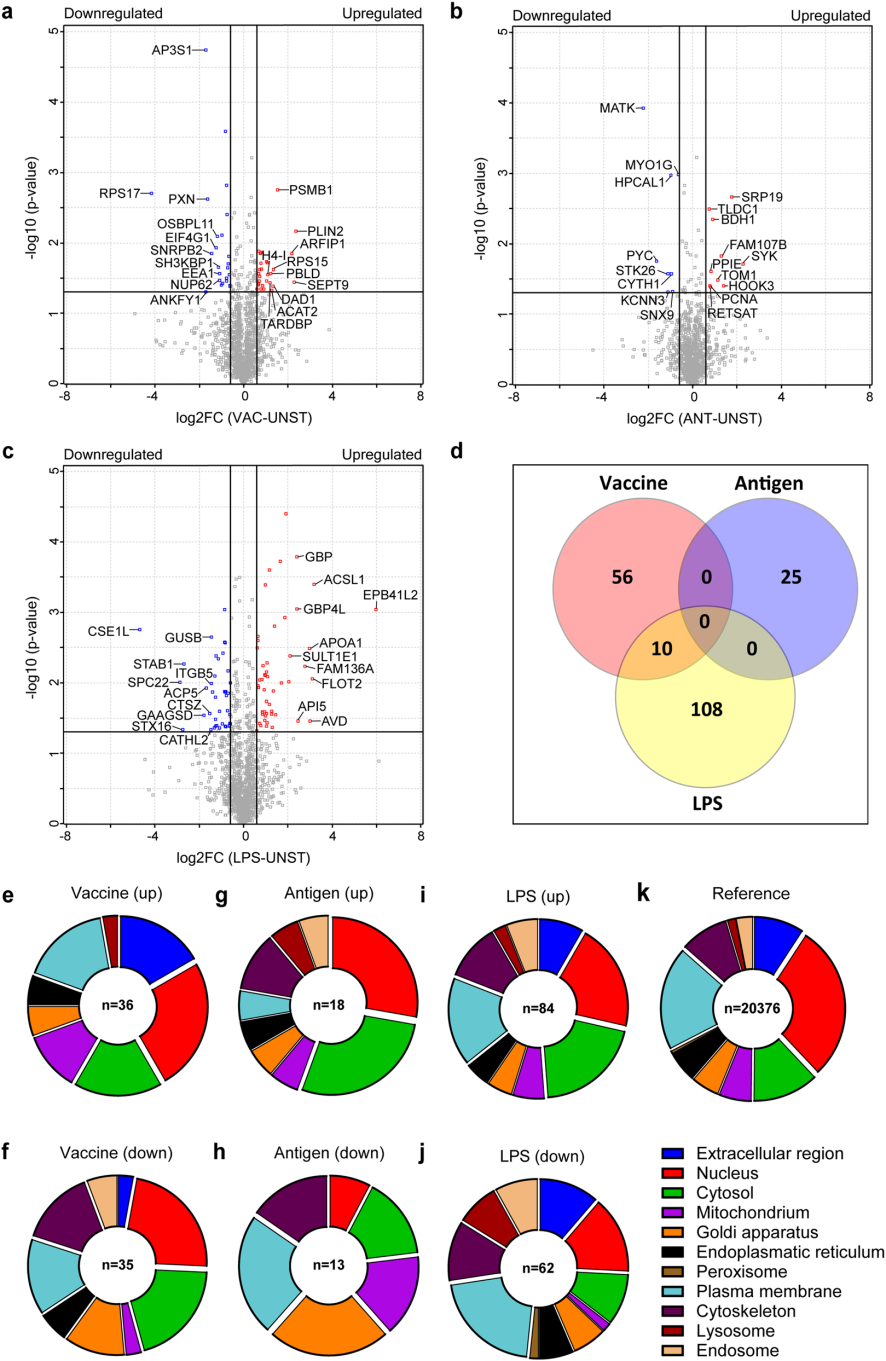
Differentially expressed proteins identified by LC–MS/MS after stimulation of chBMDCs. Volcano plots show differentially expressed proteins of chBMDCs after stimulation with LPS (**a**), an IBV + NDV vaccine (**b**) and IBV antigens only (**c**). Proteins that are ≥ 1.5-fold upregulated or downregulated and significantly different from the unstimulated controls are shown respectively in red and blue. The protein names are shown for up to 10 of the most upregulated and downregulated proteins for each condition. (**d**) A total of 199 proteins were found to be differentially expressed after the different stimulations. The overlap in differentially expressed proteins between stimulation conditions is given by a Venn diagram, which shows an overlap of 10 proteins between LPS- and vaccine-stimulated chBMDCs. The subcellular localization of differentially expressed proteins after stimulation with LPS (**e** and **f**), IBV + NDV vaccine (**g** and **h**) and IBV antigen only (**i** and **j**) was determined using gene ontology terms for cellular compartments (GOCC). A distinction was made between upregulated (**e**, **g** and **i**) and downregulated (**f**, **h** and **j**) differentially expressed proteins. The GOCC terms of all proteins from the UniProtKB reference database are shown as well (**k**). Not all proteins could be annotated with gene ontology terms for cellular compartments and some proteins were annotated with multiple entries.

Quantitative proteome analysis was performed for chBMDCs stimulated with a IBV + NDV vaccine, IBV antigen or LPS to identify the respective potential biomarkers. Initially, LC–MS/MS led to the identification of 2,828 proteins (Supplementary Table S1), 991 of which were identified multiple times, because several UniProtKB accession codes were present for these individual protein sequences, and these were manually removed based on identical expression levels in all samples. The resulting of 1837 proteins contained 153 quantitative DEPs, which were found to be differentially expressed with statistical significance (≥ 1.5-fold with *p* ≤ 0.05) after stimulation with IBV + NDV vaccine, IBV antigen or LPS (Supplementary Tables S2). In addition, 46 proteins were differentially expressed at a qualitative level, *i.e.* these proteins were detected only in either unstimulated or any of the stimulated cultures of chBMDCs (Supplementary Tables S2). We found 32 upregulated and 34 downregulated DEPs after stimulation with the IBV + NDV vaccine (Fig. 1a). Less DEPs, 14 upregulated and 11 downregulated proteins, were found after stimulation with IBV antigen (Fig. 1b). Stimulation with LPS induced a total of 66 upregulated and 52 downregulated DEPs (Fig. 1c). The Venn diagram (Fig. 1d) shows overlaps of DEPs induced by the different stimuli. The overlap between DEPs from IBV + NDV vaccine- and LPS-stimulated chBMDCs comprised 10 proteins of which five showed a change into the same direction for both stimuli (Supplementary Table S6). No DEPs were shared between IBV + NDV vaccine and IBV antigen, or LPS and IBV antigen.

The DEPs after stimulation with IBV + NDV vaccine (Figs. 1e and f), IBV antigen (Figs. 1g and h) or LPS (Figs. 1i and j) were annotated for subcellular localization using gene ontology terms for cellular compartments. The DEPs were distributed throughout all major cellular compartments. Overall, most DEPs were associated with the nucleus, plasma membrane and the cytosol, which is similar to the overall distribution of annotated proteins in the UniProtKB reference database (Fig. 1k). Compared to the reference, upregulated DEPs of vaccine-stimulated chBMDCs were relatively often associated with the extracellular region (FDR = 0.054) (Fig. 1e), whereas downregulated DEPs were more associated with the cytosol (FDR = 0.059) (Fig. 1f). Stimulation with IBV antigen resulted in a significant enrichment of upregulated DEPs associated with the cytosol (FDR = 0.046) and downregulated DEPs associated with the Golgi apparatus (FDR < 0.050) (Figs. 1g and h). LPS stimulation of chBMDCs resulted in a significantly large proportion of upregulated DEPs associated with the cytosol (FDR < 0.001) and downregulated DEPs associated with the plasma membrane (FDR = 0.043), lysosome (FDR = 0.003) and endosome (FDR = 0.037) (Figs. 1i and j).

### Differentially expressed proteins of vaccine-stimulated chBMDCs cluster in functional networks related to mRNA translation, immune responses, metabolism and the proteasome

**Figure 2:**
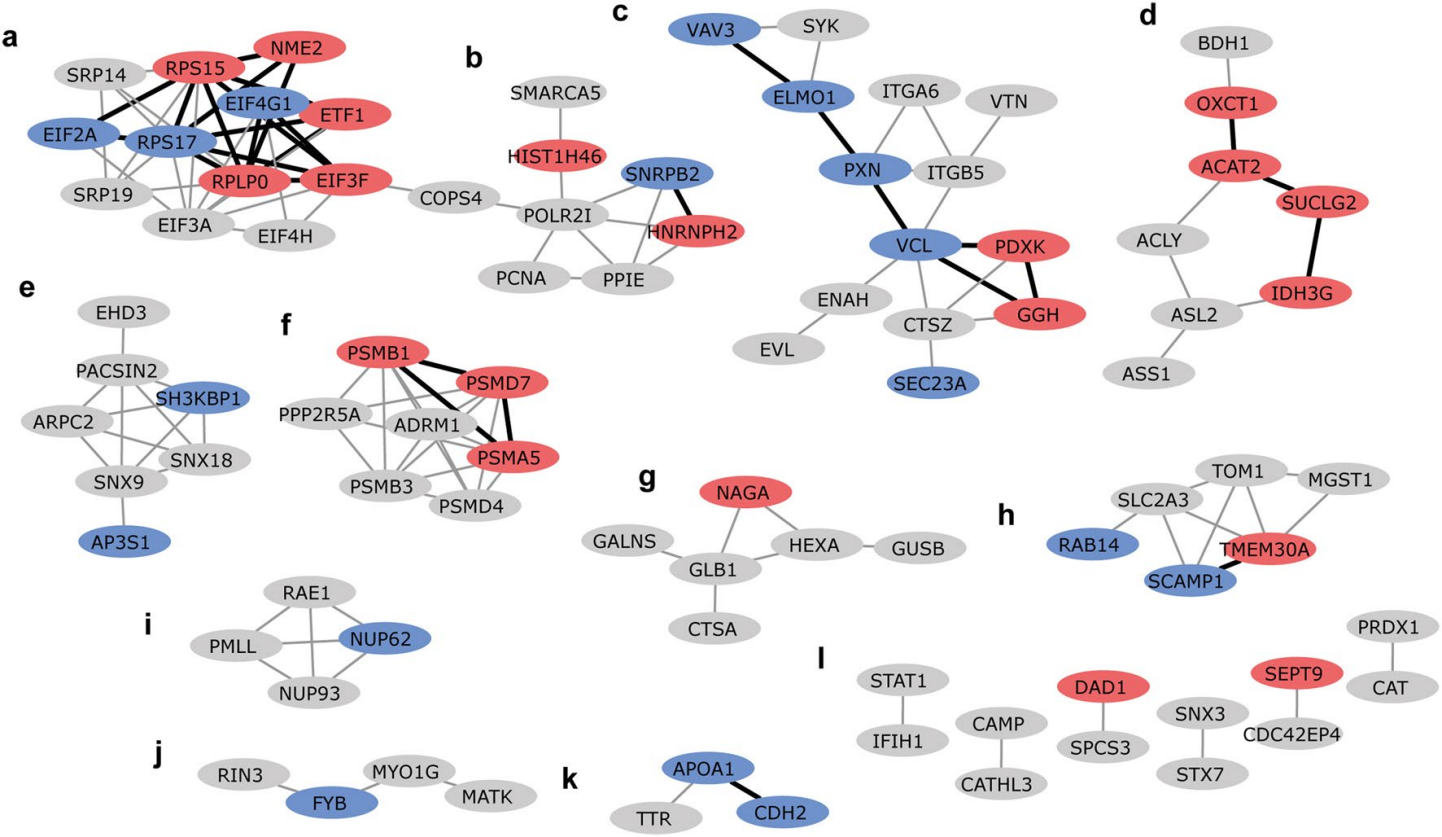
Cytoscape STRING network of differentially expressed proteins shows clusters (**a-l**) of differentially expressed proteins after IBV + NDV vaccine stimulation. Upregulated differentially expressed proteins are shown in red and downregulated differentially expressed proteins are shown in blue. Proteins in grey were not significantly differentially expressed after IBV + NDV vaccine stimulation. Networks are only shown for proteins with a minimum required interaction score of 0.700 (high confidence). STRING interactions between proteins that are both differentially expressed by vaccine exposure are shown in black, otherwise in grey. The letters indicate different clusters with cluster **l** representing six clusters comprising two proteins each.

As a next step, an enrichment analysis of protein–protein interaction networks was performed using STRING. A STRING network was created using the DEPs found for the different stimuli. From the 199 DEPs that were included in the analysis we found 91 DEPs that formed 136 high confidence physical or functional protein–protein interactions (Figs. 2, 5 and 6). These DEPs formed 16 clusters comprising 2 to 14 proteins (Supplementary Table S7).

**Figure 3:**
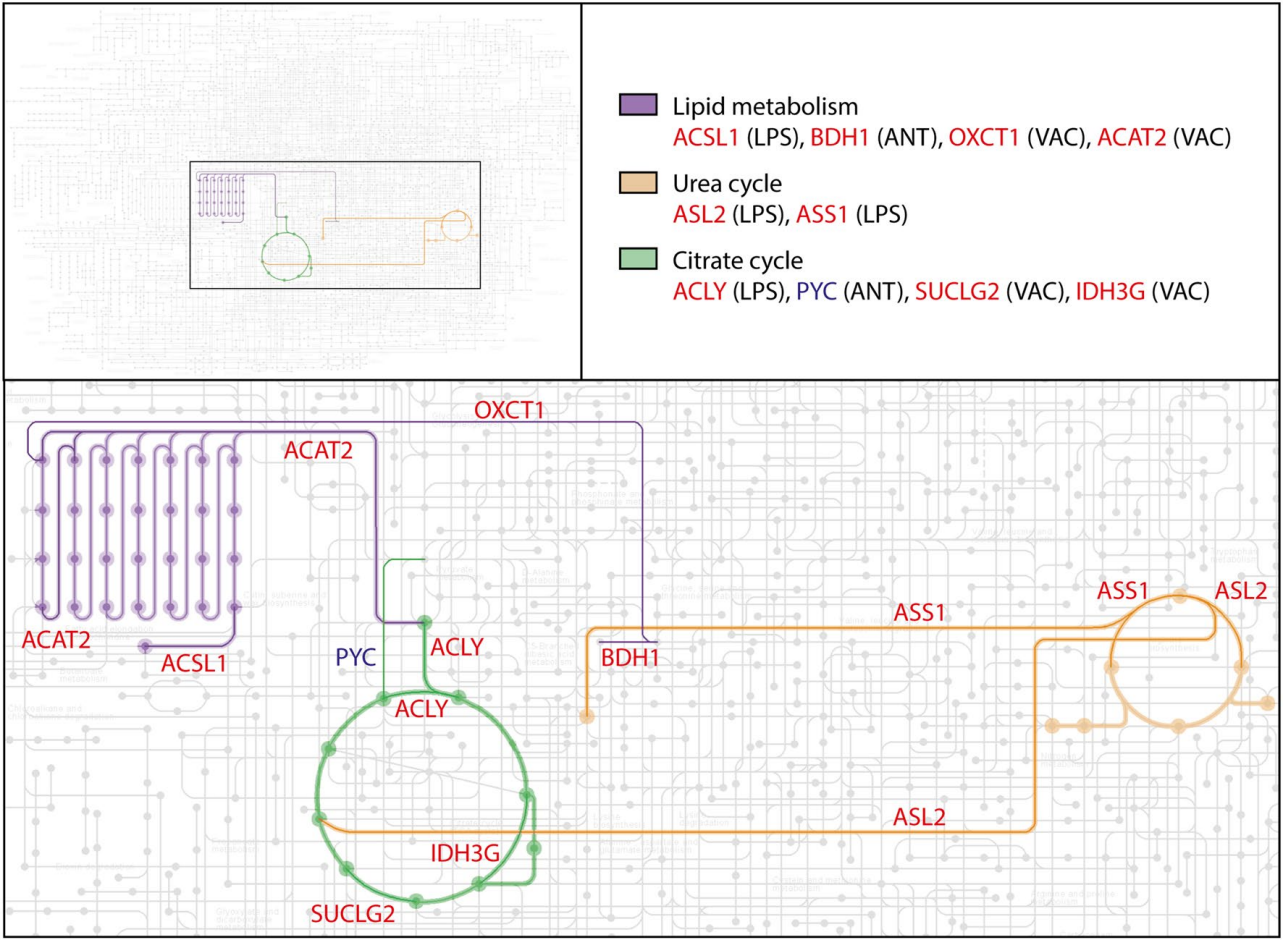
Interconnected metabolic pathways of chBMDCs change upon stimulation. The KEGG metabolic pathways map (gga01100)^29^ was modified to show differentially expressed proteins after stimulation with LPS, IBV antigen or IBV + NDV vaccine. Downregulated proteins are shown in blue and upregulated proteins are shown in red. The differentially expressed proteins could be categorized in members of lipid metabolism (purple), the urea cycle (orange) or citrate cycle (green).

**Figure 4:**
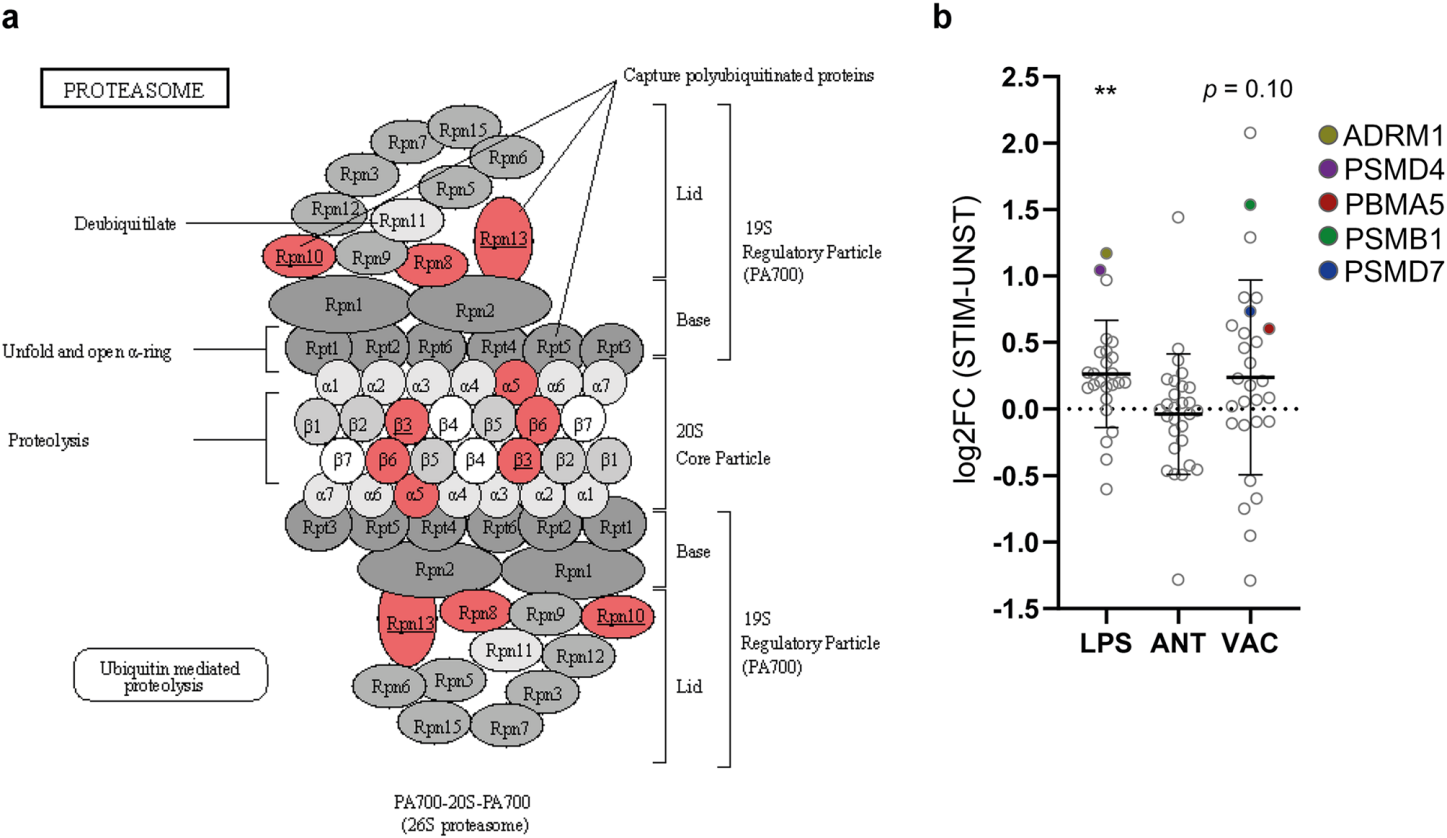
Differential expression of several proteasomal subunits in chBMDCs upon stimulation with LPS or IBV + NDV vaccine. The KEGG proteasome map (gga03050)^29^ shows upregulated proteins in red and others in grayscale (**a**). Underlined subunits PSMB3 (β3), PSMD4 (Rpn10) and ADRM1 (Rpn13) were upregulated after LPS stimulation, whereas the non-underlined subunits PSMA5 (α5), PSMB1 (β6) and PSMD7 (Rpn8) were upregulated after stimulation with IBV + NDV vaccine. Differential expression of all proteasomal subunits are shown for LPS-, IBV antigen- and IBV + NDV vaccine-stimulated chBDMCs (**b**). Quantitative DEPs are indicated by different colors, except for PSMB3, which was identified as qualitative DEP. Statistically significant differences between stimulated and unstimulated chBMDCs are indicated by ** for *p* < 0.01.

The DEPs of IBV + NDV vaccine-stimulated chBMDCs were found overrepresented in clusters a (8/12), c (7/14), d (4/8) and h (3/6) (Fig. 2). For cluster a, the top ranking functional enrichments were “mRNA translation” (Reactome: GGA-72766, FDR = 1.6 × 10^–12^; GO: 0,006,412, FDR = 2.3 × 10^–4^) representing all proteins except NME2, an enzyme required for synthesis of nucleoside triphosphates, and ETF1, which plays a role in the termination of translation. Cluster a is linked to cluster b through COPS4, a subunit of the COP9 signalosome complex. After stimulation with IBV + NDV vaccine, 3/7 proteins of cluster b were identified as DEPs. The highest ranking functional enrichments for cluster b were “DNA repair” (Reactome: GGA-73894, FDR = 1.3 × 10^–8^), “transcription-coupled nucleotide excision repair [TC-NER]” (Reactome: GGA-6781827, FDR = 7.9 × 10^–7^) and mRNA splicing (Reactome: GGA-72163, FDR = 6.5 × 10^–6^), which together comprised all DEPs of this cluster. Cluster c was most functionally enriched for “immune system” (Reactome: GGA-168256; FDR = 4.5 × 10^–8^), followed by the more specific description “innate immune system” (Reactome: GGA-168249; FDR = 2.7 × 10^–7^). Except for PXN, which is involved in focal adhesion, all DEPs found in cluster c after IBV + NDV vaccine stimulation were described to be involved in immune responses. Within cluster d, two upregulated DEPs (OXCT1 and ACAT2) are part of lipid metabolism and another two upregulated DEPs (SUCLG2 and IDH3G) are part of the citrate cycle (Fig. 3). Cluster f was functionally enriched for “proteasome” (KEGG: gga03050; FDR = 6.4 × 10^–12^) and comprised subunits of this protein complex that has a critical role in cellular proteolysis, including in the processing of antigens for presentation on the cell surface to the adaptive immune system. Proteins of cluster f were mapped to the KEGG proteasome pathway (Fig. 4a), which demonstrated that two upregulated DEPs of chBMDCs stimulated with IBV + NDV vaccine, PSMA5 (α5) and PSMB1 (β6), are part of the proteasomal 20S core particle. The third DEP, PSMD7 (Rpn8), is part of the Rpn8-Rpn11 heterodimer with a function in removal of polyubiquitin tags before protein degradation in the proteasome^30^. A proteasomal subunit in close proximity to the Rpn8-Rpn11 heterodimer is PSMD13 (Rpn9), which showed a trend towards upregulation (fold change = 4.2; *p* = 0.07). Finally, the polyubiquitin-capturing protein PSMD4 (Rpn10) also showed a trend towards downregulation (fold change = 1.6; *p* = 0.07). Overall, expression of proteasomal subunits chBMDCs were increased by 20% (*p* = 0.003) and 18% (*p* = 0.10) after stimulation with LPS and IBV + NDV vaccine, respectively (Fig. 4b).

Besides the proteins of cluster c, more proteins that were differentially expressed by chBMDCs after stimulation with IBV + NDV vaccine were found to be part of the Reactome “immune system” pathways (GGA-168256). This includes the proteasomal proteins of cluster f, but also BLMH, FYB, RAB14, TCEB1, SCAMP1 and MIF. Although the “immune system” annotation was not assigned to IFITM3, this protein has a known role in antiviral immune responses^31^.

### The small number of differentially expressed proteins of antigen-stimulated chBMDCs showed limited clustering

**Figure 5:**
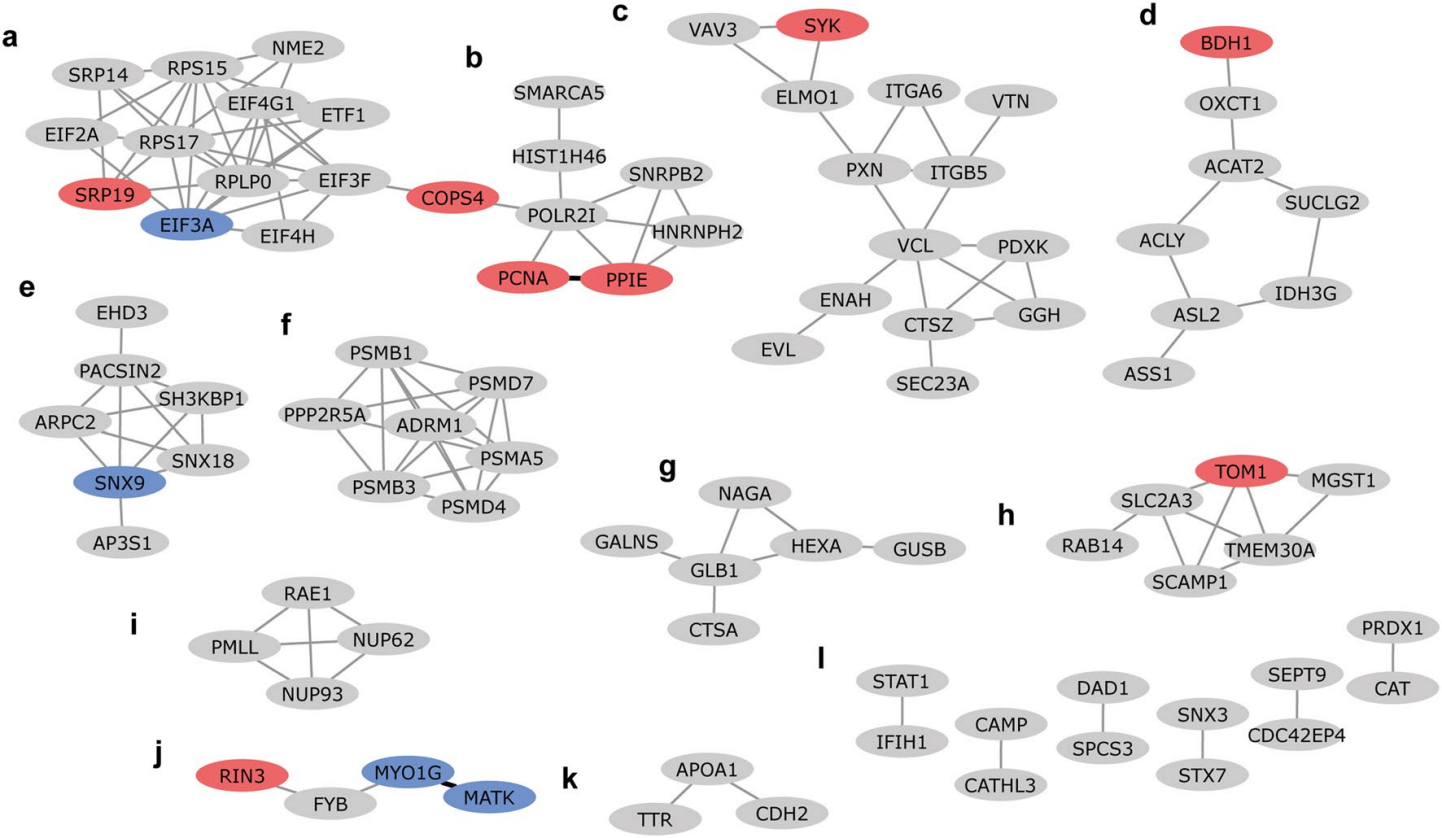
Cytoscape STRING network of differentially expressed proteins shows clusters (**a-l**) of differentially expressed proteins after IBV antigen stimulation. Upregulated differentially expressed proteins are shown in red and downregulated differentially expressed proteins are shown in blue. Proteins in grey were not significantly differentially expressed after IBV antigen stimulation. Networks are only shown for proteins with a minimum required interaction score of 0.700 (high confidence). STRING interactions between proteins that are both differentially expressed by antigen exposure are shown in black, otherwise in grey. The letters indicate different clusters with cluster **l** representing six clusters comprising two proteins each.

A limited number of DEPs was found for IBV antigen-stimulated chBMDCs. The only cluster with a majority of DEPs after stimulation with IBV antigen was cluster j (Fig. 5). This cluster was annotated for Src homology 2 (SMART: SM00252; FDR = 1.5 × 10^–3^) and 3 (SMART: SM00326; FDR = 3.2 × 10^–3^) domains. The DEPs RIN3, MATK and SYK possess a Src homology 2 domain. Apart from this, IBV antigen seemed to have little impact on functional pathways of chBMDCs.

### Differentially expressed proteins of LPS-stimulated chBMDCs cluster in functional networks related to immune responses, metabolism, endocytosis, the proteasome and lysosomal enzymes

**Figure 6:**
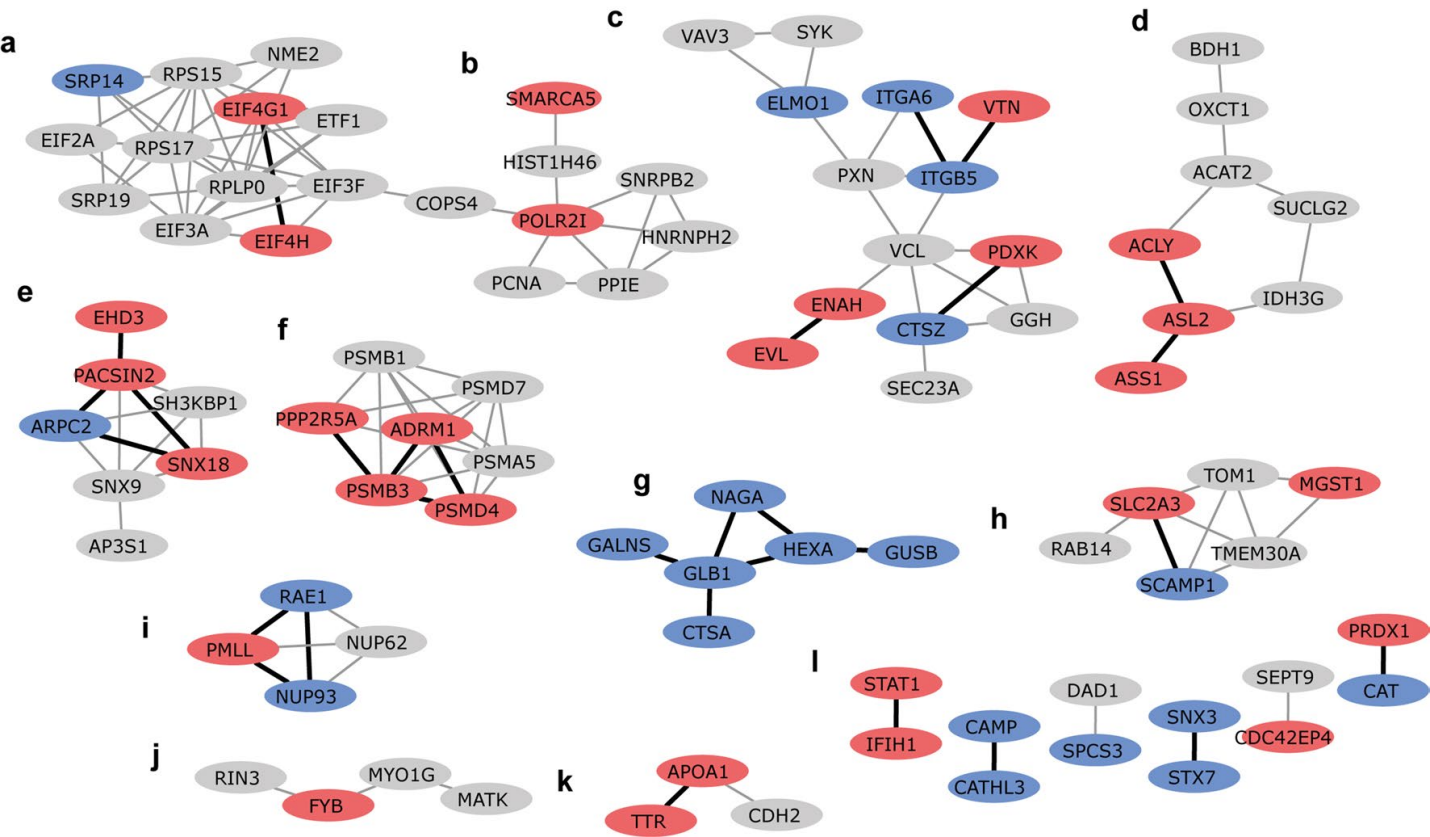
Cytoscape STRING network of differentially expressed proteins shows clusters (**a-l**) of differentially expressed proteins after LPS stimulation. Upregulated differentially expressed proteins are shown in red and downregulated differentially expressed proteins are shown in blue. Proteins in grey were not significantly differentially expressed after LPS stimulation. Networks are only shown for proteins with a minimum required interaction score of 0.700 (high confidence). STRING interactions between proteins that are both differentially expressed by LPS exposure are shown in black, otherwise in grey. The letters indicate different clusters with cluster **l** representing six clusters comprising two proteins each.

The DEPs of LPS-stimulated chBMDCs were found overrepresented in clusters c (8/14), d (4/8), e (4/7), f (4/7), g (6/6), h (3/6) and i (3/4) (Fig. 6). Similar to stimulation with IBV + NDV vaccine, cluster c contained many DEPs after stimulation with LPS. Within this cluster, two DEPs (ELMO1 and PDXK) were shared between IBV + NDV vaccine- and LPS-stimulated chBMDCs. Furthermore, “regulation of actin cytoskeleton” (KEGG: gga04810; FDR = 3.3 × 10^–6^) and “focal adhesion” (KEGG: gga04510; FDR = 3.3 × 10^–6^) were found as important functional enrichments. All proteins of cluster d were part of “metabolic pathways” (KEGG: gga01100; FDR = 4.6 × 10^–7^), more specifically they were part of the citrate cycle, urea cycle or fatty acid metabolism (Fig. 3). Cluster e was functionally enriched for proteins containing “Src homology 3 domains” (PFAM: PF14604; FDR = 2.2 × 10^–6^), “clathrin-mediated endocytosis” (Reactome: GGA-8856828; FDR = 1.6 × 10^–6^) and “membrane trafficking” (Reactome: GGA-199991; FDR = 4.6 × 10^–6^). Similar to stimulation with IBV + NDV vaccine, cluster f contained DEPs after stimulation with LPS, including the non-ATPase regulatory proteasomal subunits PSMD4 (Rpn10) and ADRM1 (Rpn13) with a function in capturing polyubiquitinated proteins for proteolysis (Fig. 4). A third proteasomal subunit, PSMC3 (Rpt5), with a function in the capture of polyubiquitinated proteins showed a trend of being upregulated after LPS stimulation (fold change = 1.3; p = 0.053). The peptide transporter TAP2, which is responsible for the translocation of proteasome-processed peptides into the endoplasmatic reticulum for loading on MHC molecules, was found upregulated after LPS stimulation (Supplementary Table S2). Cluster g comprised lysosomal enzymes (KEGG: gga04142; FDR = 8.4 × 10^–13^) and was completely downregulated after LPS stimulation. In addition to the proteins of cluster g, other lysosomal enzymes were downregulated upon LPS stimulation (Supplementary Figure 2), including GAAGSD, DNaseII, ACP5, and SMPD1. Furthermore, the minor lysosomal membrane protein NPC was downregulated. Cluster h was functionally enriched for “organic substance transport” (GO: 0,071,702; FDR = 1.7 × 10^–5^) and “neutrophil degranulation” (Reactome: GGA-6798695; FDR = 4.8 × 10^–5^). Cluster i comprised four proteins that were found to be related to small ubiquitin-related modifier (SUMO) E3 ligases (Reactome: GGA-3108232; FDR = 1.1 × 10^–8^).

### Gene expression analysis shows partial overlap with mass spectrometry data, resulting in candidate vaccine-associated biomarkers PSMB1 and PLIN2

**Figure 7:**
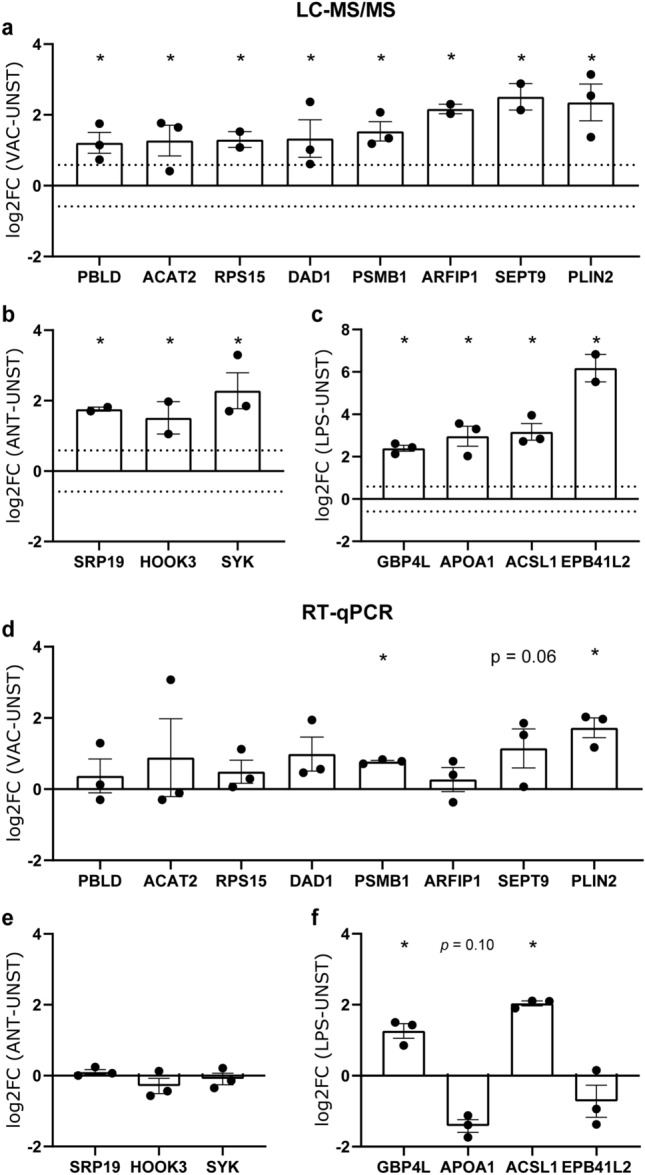
Comparison of protein and gene expression levels of differentially expressed proteins found to be most upregulated in mass spectrometry. Differentially expressed proteins of (**a**) IBV + NDV vaccine-, (**b**) IBV antigen- (**c**) and LPS-stimulated chBMDCs were selected for gene expression analysis using qPCR (resp. **d–f**). The expression levels of proteins and genes were analyzed for three independent replicates. The error bars show the SEM, and the dotted lines represent the cut-off values of 1.5-fold change that were used to select differentially expressed proteins in mass spectrometry. Statistically significant differences between stimulated and unstimulated chBMDCs are indicated by * for *p* < 0.05.

Finally, we set out to investigate whether any of the DEPs could also be detected as differentially expressed genes (DEGs) by RT-qPCR, a method that could directly be used to evaluate biomarker expression in chickens immune cells. Similar to the proteomics data, chBMDCs were stimulated for 24 h with IBV + NDV vaccine, IBV antigen or LPS. The eight most significantly upregulated DEPs of IBV + NDV vaccine-stimulated chBMDCs were selected to evaluate gene expression levels (Fig. 7a). Moreover, three DEPs were selected for IBV antigen-stimulated chBMDCs (Fig. 7b) and four DEPs for LPS-stimulated chBMDCs (Fig. 7c). Analysis of gene expression showed that the three out of eight tested vaccine-related DEPs were also found upregulated at the mRNA expression level, including PSMB1 (*p* = 0.042) and PLIN2 (*p* = 0.002), which were significantly upregulated, (Fig. 7d) and SEPT9, which tended to be upregulated (*p* = 0.055). None of the selected DEPs of antigen-stimulated chBMDCs were differentially expressed at the mRNA level (Fig. 7e). Two out of four DEPs of LPS-stimulated chBMDCs, GBP4L and ACSL1, were found as upregulated DEGs (Fig. 7f). Analyzing mRNA expression levels of all DEP after 8 h rather than 24 h stimulation showed an upregulation of PLIN2 (1.96-fold) by vaccine-stimulated chBMDCs, an upregulation of GBP4L (8.5-fold) and ACSL1 (9.8-fold) by LPS-stimulated chBMDCs, and a downregulation of EPB41L2 (3.0-fold) by LPS-stimulated chBMDCs, while other genes were unaffected (*data not shown*).

## Discussion

To our best knowledge, this study was the first attempt to discover biomarkers for inactivated poultry vaccines by investigating vaccine-induced immune responses with proteomics. To do so, we stimulated chBMDCs with an inactivated IBV + NDV poultry vaccine containing a mineral oil adjuvant or inactivated IBV antigen only and we analyzed the cell lysates by LC–MS/MS, which resulted in a list of DEPs that were also evaluated for mRNA expression levels using RT-qPCR. Furthermore, chBMDCs were stimulated with *E. coli* LPS as a strong stimulator of innate immune cells, as demonstrated in previous studies^14–19,32,33^, and was used to validate our methodology and to generate new insights in cellular processes involved in LPS driven maturation of chBMDCs.

Mass spectrometry analysis led to the identification of 118 DEPs in chBMDCs after stimulation with LPS, 66 DEPs after stimulation with IBV + NDV vaccine and 25 DEPs after stimulation with IBV antigen. For each stimulus, 20–25% of the DEPs were found to be uniquely expressed in either the unstimulated group or any of the stimulated groups, whereas the protein expression levels were absent or below the detection limit of the mass spectrometer in the other groups. Although statistics could not be performed for these proteins, the qualitative differences were still considered to be of practical significance. In the STRING analysis, DEPs based on qualitative differences made up 23% and were therefore not over- or underrepresented compared to DEPS based on quantitative differences. Moreover, DEPs based on qualitative differences were randomly distributed among the clusters of the STRING network (Supplementary Table S7). Therefore, inclusion of DEPs based on qualitative differences strengthened our STRING enrichment analysis without significantly changing the clusters observed.

Stimulation of chBMDCs with *E. coli* LPS resulted in most DEPs, which were associated with the (innate) immune system, regulation of the cytoskeleton, metabolic pathways, endocytosis, the lysosome, the proteasome, and SUMO E3 ligases. A previous study reported in vivo effects of LPS at the transcriptomic level^34^, showing increased transcription of genes associated with inflammatory responses, regulation of the cytoskeleton and cell migration in the bursa of Fabricius, a lymphoid organ only found in birds. This corresponds with the DEPs of the current study, annotated for functions in the innate immune system and regulation of the cytoskeleton. Moreover, many DEPs of LPS-stimulated chBMDCs, including proteins with functions in the innate immune system, have previously been described to be significantly increased at the gene expression level in the chicken caecum and spleen upon bacterial infection with *S. enteritidis*^35,36^. Finally, the SUMO E3 ligase PMLL was upregulated by LPS stimulation, which is in agreement with a study about the mammalian homologue PML that showed its importance for immune cell activation by LPS^37^.

The results of this study shed light on mechanistic aspects of LPS stimulation that result in chBMDC maturation^14–19,32,33^. First of all, increased expression of STAT1 was observed, in agreement with its requirement for DC maturation^38^. Secondly, we observed a strong downregulation of lysosomal hydrolases after LPS stimulation, in line with previous reports showing a decreased uptake of antigens by endocytosis or phagocytosis after LPS stimulation^14^. This effect is in accordance with studies in human and murine BMDCs showing reduced recruitment of lysosomal hydrolases to phagosomes, reduced phagosomal acidification and reduced phagolysosomal activity that together preserve exogenous antigens for (cross-)presentation by mature DCs^39–42^. Thirdly, we have observed an upregulation of the non-ATPase regulatory subunits PSMD4 and ADRM1 of the proteasome. The increased expression of regulatory proteasomal subunits after LPS stimulation has been reported to facilitate more efficient antigen processing in murine BMDCs^42^. The downregulation of lysosomal activity, the upregulation of proteasomal activity and the upregulation of TAP2, which is required for the transport of proteasome-processed peptides to the endoplasmic reticulum for loading on MHC molecules, suggest that antigen-presentation by chBMDCs increased as a consequence of DC maturation after LPS stimulation, similar to murine BMDCs^40,42^.

Several DEPs of LPS-stimulated chBMDCs are involved in antimicrobial activity, even though these proteins did not cluster into one STRING network. Four of these proteins were upregulated metabolic enzymes involved in the production of inflammatory mediators, namely ASL2, ASS1, IRG1 and ACSL1. The DEPs ASL2 and ASS1 are involved in nitric oxide production^43^, ACSL1, has a role in the synthesis of the inflammatory mediator prostaglandin E2^44^, and IRG1 produces itaconate^45^, all of which are inflammatory mediators with antimicrobial effects. IRG1^42^ and ACSL^46^ were previously shown to be upregulated in murine BMDCs stimulated with LPS or bacteria. Furthermore, the upregulated DEPs AVD and GBP4L have direct antimicrobial effects^45^. GBP4L was also found as DEG by RT-qPCR and is involved in the activation of innate immune cells by LPS and bacteria^47^. In contrast, the antimicrobial cathelicidins CATHL2 and CATHL3, known to neutralize LPS^48,49^, were found downregulated in the chBMDCs. Taken together, our proteomics data showed that LPS stimulation induced changes associated with cellular processes including DC maturation and antimicrobial activity that have not been described before at the protein level for chicken immune cells. In addition to its function as a strong immunostimulatory control, thereby verifying our methodology, LPS stimulation led to increased insight in relevant changes in protein expression in chBMDCs.

For IBV antigen-stimulated chBMDCs, a limited number of DEPs was observed. Of these, SYK was the strongest upregulated DEP. SYK was previously shown to be upregulated in response to uric acid crystals in humans^50^. Uric acid crystals are also present in the IBV antigens, since these are dissolved in allantoic fluid from embryonated chicken eggs^51^. Previously, we have shown that allantoic fluid has immunostimulatory properties, potentially through the presence of uric acid crystals, leading to increased Fc receptor-mediated phagocytosis in macrophages^52^, in which SYK is likely to be involved^53^.

The STRING analysis for vaccine-stimulated chBMDCs showed that the DEPs identified were largely related to the immune response, the proteasome, metabolism, and mRNA translation. Since there was no overlap between chBMDCs stimulated with the inactivated IBV antigen and those that were stimulated with vaccine, the effects of the vaccine were likely to be induced by either the mineral oil adjuvant or inactivated NDV antigen as the other components of the bivalent vaccine. Due to its slow metabolization, mineral oil acts as a depot that releases antigen over a long period of time, during which strong humoral immune responses can be developped^54–58^. Some upregulated DEPs of vaccine-stimulated chBMDCs involved in the storage (PLIN2 and TMEM23A) and metabolism (LIPA, OCXT1 and ACAT1) of lipids, or part of the citric acid cycle (SUCLG2 and IDH3G), may together contribute to the metabolism of internalized mineral oil adjuvant and hence to the release of antigen in course of time. Two more upregulated proteins involved in lipid storage, SYAP1 and RAB18, were very close to significance (*p* < 0.06). In contrast, OSBP11L and APOA1 involved in lipid transport were found as downregulated DEPs. The storage of lipids in IBV + NDV-stimulated chBMDCs is in agreement with microscopic observations (Supplementary Figure 3d). Lipid droplets have been described as important for the induction of cross-presentation by saponin-based adjuvants^59^. PLIN2 was also found as a DEG and might therefore be particularly useful as a biomarker of the mineral oil-adjuvanted vaccine since its expression can be evaluated with RT-qPCR in addition to mass spectrometry.

Studies in mice suggest that water-in-oil-adjuvanted vaccines induce the release of damage-associated molecular patterns (DAMPs) by stressed or damaged cells, which promotes the differentiation of follicular helper T cells^60^ and the induction of humoral adaptive immune responses^61^. In our present study, we found significantly DEPs in chBMDCs stimulated with the IBV + NDV vaccine involved in the innate immune response. The DEPs VAV3 and ELMO1, both involved in the formation of phagocytic cups, were downregulated, which fits to the functional change of DCs, from uptake of antigens to presentation of antigens, during DC maturation. Moreover, the proteasomal subunits PSMA5, PSMB1 and PSMD7 play a role in antigen processing and were found significantly upregulated. Of these, PSMB1 was also found as an upregulated DEG. In addition, the upregulated DEP BLMH has been implicated in antigen-processing for presentation on MHC-I molecules, downstream of the proteasome^62^. Finally, the pro-inflammatory cytokine MIF, which acts as a DAMP^63^, was found to be significantly upregulated in response to the vaccine.

The aim of this study was to identify protein targets to be used for in vitro quality assessment of inactivated poultry vaccines and to generate new insights in aspects of the mechanisms-of-action by which these vaccines as well as LPS activate chicken DCs. Stimulation with LPS led to decreased expression of DEPs associated with phagolysosomal activity, thereby preserving exogenous antigens for (cross-)presentation, and to increased expression of DEPs associated with proteasomal antigen processing, which together suggests that antigen presentation increases during chBMDC maturation. Furthermore, LPS stimulation led to an increase in DEPs associated with antimicrobial activity. The DEPs of vaccine-stimulated chBMDCs were found largely associated with mRNA translation, immune responses, lipid metabolism and proteasomal degradation, which provides new insights about the effects that inactivated poultry vaccines have on chicken DCs. Furthermore, two significantly upregulated DEPs of vaccine-stimulated chBMDCs included PLIN2, involved in the storage of lipid droplets, and PSMB1, a proteasomal subunit, and were also found as upregulated DEGs using RT-qPCR. Future studies should include different conforming as well as non-conforming vaccine batches to determine whether the identified DEPs and DEGs are able to discriminate between batches of good and suboptimal quality. The identification of these vaccine-associated biomarkers may facilitate the transition from in vivo to cell-based quality assessment of inactivated poultry vaccines.

## Supplementary Information

Supplementary Information 1.

Supplementary Information 2.

Supplementary Information 3.

Supplementary Information 4.

Supplementary Information 5.

Supplementary Information 6.

Supplementary Information 7.

Supplementary Information 8.

Supplementary Information 9.
