# Supplementary material for: Proteomic analysis of chicken bone marrow-derived dendritic cells in response to an inactivated IBV + NDV poultry vaccine
Source: Sci Rep. 2021 Jun 16;11:12666. doi: 10.1038/s41598-021-89810-3 (PMC8209092; doi:10.1038/s41598-021-89810-3)
Supplement: Supplementary file 5 — Supplementary Information 5. [file 41598_2021_89810_MOESM5_ESM.docx]

**Supplementary Table S5** Primer sequences for RT-qPCR to assess gene expression of selected upregulated DEPs.

| **Gene** | **Reference** | **Type** | **Sequences (5’-3’)** |
| --- | --- | --- | --- |
| PBLD | ENSGALT00000006268.6 | Forward | CGGTTTGTCTGCTCGAAAATGAC |
|  |  | Reverse | AACCATCGGAGTCCAAAGCG |
| ACAT2 | ENSGALT00000019060.5 | Forward | CCGGATCGTATCTTGGGCTCA |
|  |  | Reverse | AGTCCAGCCGGCTTTCTCAATA |
| RPS15 | ENSGALT00000070660.2 | Forward | CCAAGATGGCAGAAGTGGAGCA |
|  |  | Reverse | AGGACATATCGAGCAGCTGGT |
| DAD1 | ENSGALT00000048785.2 | Forward | AGCTTCATCCTCGGCGTTTGT |
|  |  | Reverse | CCCTCTCTGGTGAAATGCCTTGG |
| PSMB1 | ENSGALT00000018217.5 | Forward | GCCATTTTGCCGTGAGACAGC |
|  |  | Reverse | ACACAGTCCCGCCGTTGAAG |
| ARFIP1 | ENSGALT00000065974.3 | Forward | AGGTTCCTCAGCATCACCCA |
|  |  | Reverse | ACCGGTCCACTCTTCTGCTG |
| SEPT9 | ENSGALT00000047767.2 | Forward | GAAGGTCCAGAGGCCGTTCC |
|  |  | Reverse | GCATCGATCCCCACGTAGCC |
| PLIN2 | ENSGALT00000083439.2 | Forward | CCGCTCAACTGGCTGGTTCC |
|  |  | Reverse | GGGCCATTTGTGCGTGAAAAGT |
| SRP19 | ENSGALT00000000294.6 | Forward | GGTAGGCCTGGTCGTTAGCA |
|  |  | Reverse | AAATCTCTCCTTGTCGGCCGG |
| HOOK3 | ENSGALT00000024828.6 | Forward | ACAACCATGAGATTCTAGGGCAAC |
|  |  | Reverse | GCATTCTTCCAAGCTCCGCA |
| SYK | ENSGALT00000032807.5 | Forward | CCCTCTGGCAGTTAGTTGAGCA |
|  |  | Reverse | TCACTTTCTGAGCCATGCCGT |
| GBP4L | ENSGALT00000069561.2 | Forward | AGCGGCCCATCAGTGAGGAT |
|  |  | Reverse | GAGCCGCGTCCCATTGTAGT |
| APOA1 | ENSGALT00000011524.6 | Forward | CTGTGCTCTTCCTGACGGG |
|  |  | Reverse | GAGCCATGTCCTCACGCA |
| ACSL1 | ENSGALT00000017294.4 | Forward | AGCCCAGGGAGAGTACATAGCA |
|  |  | Reverse | AGGCCTGCAAGCTCTCTCCA |
| EPB41L2 | XM_025148931.1 | Forward | CATCGGGCCGCTAAGAGGTT |
|  |  | Reverse | TGTCGTGTCTGTGCTTGCGT |
| GAPDH | ENSGALT00000023323.6 | Forward | GTGGTGCTAAGCGTGTTATC |
|  |  | Reverse | GCATGGACAGTGGTCATAAG |
