# Supplementary material for: Proteomic analysis of chicken bone marrow-derived dendritic cells in response to an inactivated IBV + NDV poultry vaccine
Source: Sci Rep. 2021 Jun 16;11:12666. doi: 10.1038/s41598-021-89810-3 (PMC8209092; doi:10.1038/s41598-021-89810-3)
Supplement: Supplementary file 8 — Supplementary Information 8. [file 41598_2021_89810_MOESM8_ESM.pdf]

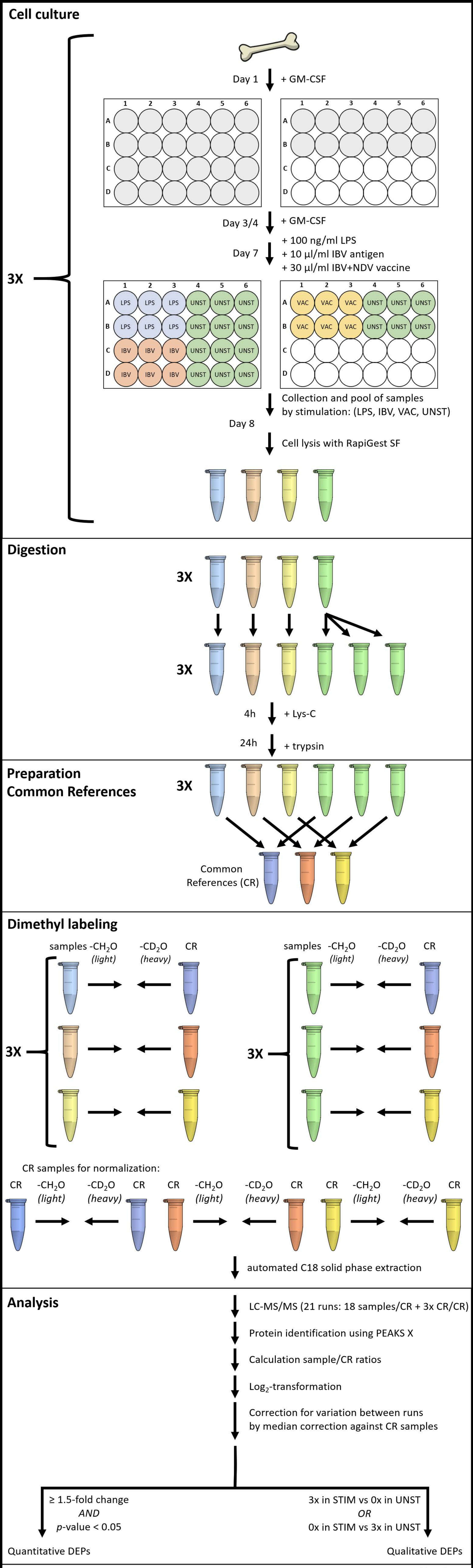

Supplementary Figure 1 Graphical overview of the experimental workflow. This figure graphically summarizes the experimental workflow as described in more detail in the Methods section.
