# Supplementary material for: Proteomic analysis of chicken bone marrow-derived dendritic cells in response to an inactivated IBV + NDV poultry vaccine
Source: Sci Rep. 2021 Jun 16;11:12666. doi: 10.1038/s41598-021-89810-3 (PMC8209092; doi:10.1038/s41598-021-89810-3)
Supplement: Supplementary file 9 — Supplementary Information 9. [file 41598_2021_89810_MOESM9_ESM.docx]

**Proteomic analysis of chicken bone marrow-derived dendritic cells in response to an inactivated IBV+NDV poultry vaccine**

**Running title:** Proteomic analysis inactivated poultry vaccines

Robin H. G. A. van den Biggelaar^1^, Larissa van der Maas^2^, Hugo D. Meiring^2^, Jeroen L.A. Pennings^3^, Willem van Eden^1^, Victor P. M. G. Rutten^1,4^, Christine A. Jansen^1,5,*^

^1^Division of Infectious Diseases and Immunology, Department of Biomolecular Health Sciences, Faculty of Veterinary Medicine, Utrecht University, the Netherlands

^2^Intravacc (Institute for Translational Vaccinology), the Netherlands

^3^Centre for Health Protection, National Institute for Public Health and the Environment (RIVM), the Netherlands

^4^Department of Veterinary Tropical Diseases, Faculty of Veterinary Science, University of Pretoria, South Africa

^5^Cell Biology and Immunology Group, Department of Animal Sciences, Wageningen University and Research, Wageningen, the Netherlands

*email: c.a.jansen@uu.nl

# Supplementary Figures


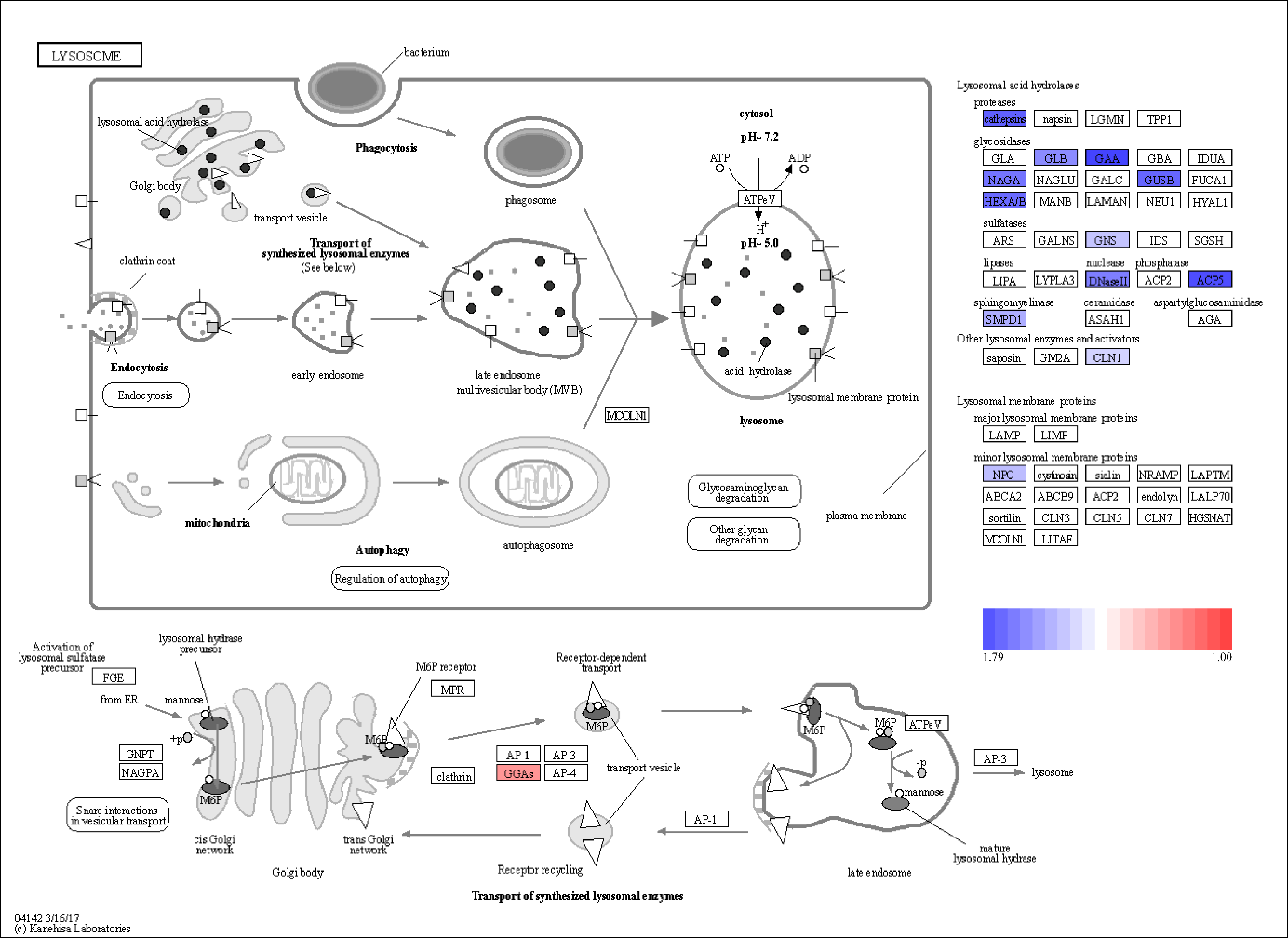


**Supplementary Figure 2 Stimulation of chBMDCs with LPS led to the significant downregulation of lysosomal enzymes.** The KEGG lysosome map (gga04142)^29^ shows differentially expressed proteins of LPS-stimulated chBMDCs in the lysosome. Downregulated proteins are shown in blue and upregulated proteins are shown in red.

**
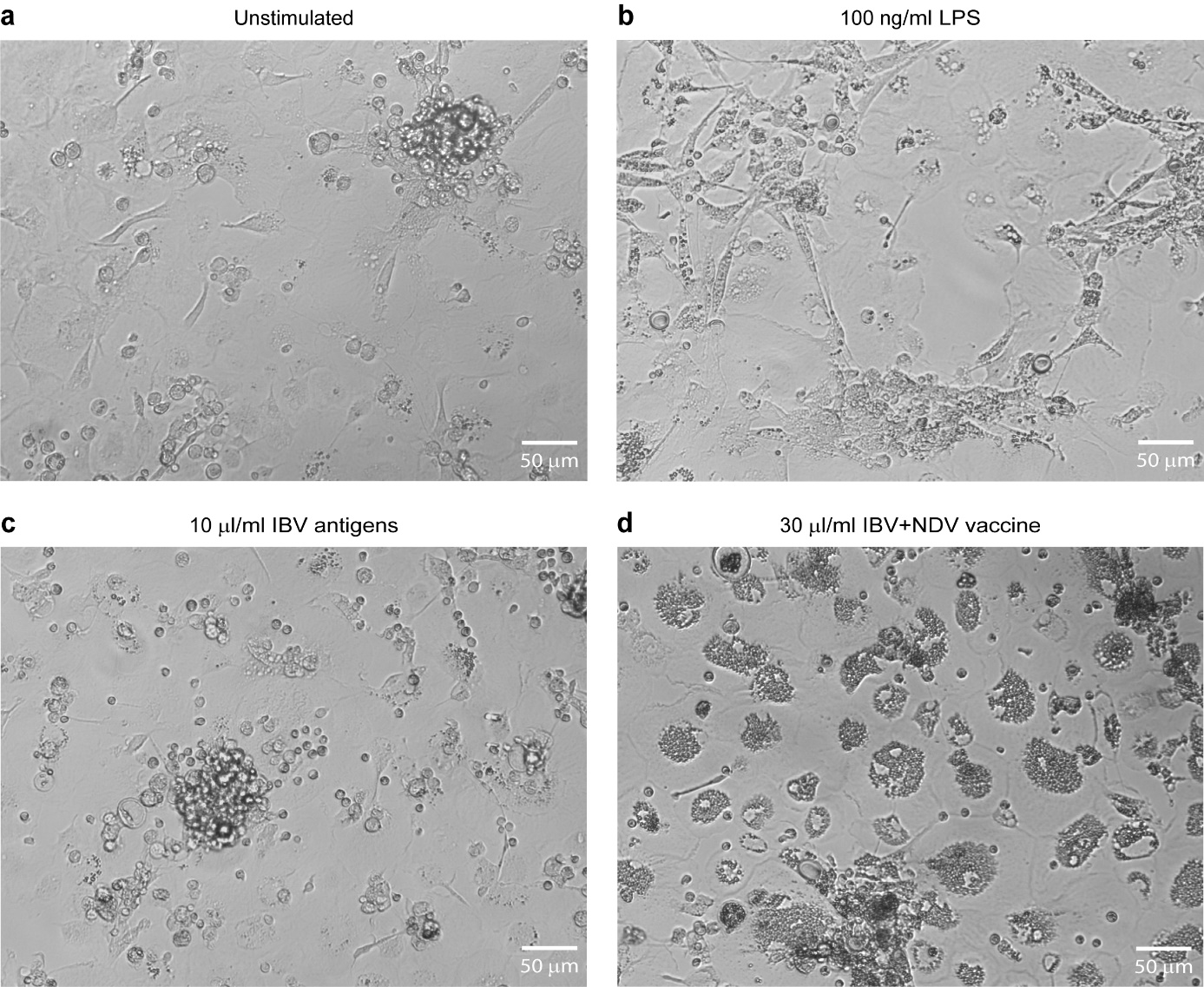
**

**Supplementary Figure 3 The morphology of chBMDCs changes upon stimulation.** The chBMDC cultures were left unstimulated (**a**) or were stimulated with 100 ng/ml LPS (**b**), 10 μl/ml IBV antigen (**c**) or 30 μl/ml IBV+NDV vaccine (**d**). Subsequently, the cell morphology of the cultures was captured with brightfield light microscopy at 100x magnification.
